# Viral, Serological, and Antioxidant Investigations of Equine Rhinitis A Virus in Serum and Nasal Swabs of Commercially Used Horses in Poland

**DOI:** 10.1155/2018/8719281

**Published:** 2018-04-22

**Authors:** Barbara Bażanów, Agnieszka Frącka, Natalia Jackulak, Ewa Romuk, Tomasz Gębarowski, Aleksander Owczarek, Dominika Stygar

**Affiliations:** ^1^Faculty of Veterinary Medicine, Department of Pathology, Division of Microbiology, Wrocław University of Environmental and Life Sciences, Wrocław, Poland; ^2^Department of Biochemistry, School of Medicine with Dentistry Division in Zabrze, Medical University of Silesia, Katowice, Poland; ^3^Department of Basic Medical Sciences, Wrocław Medical University, Wrocław, Poland; ^4^Department of Statistics, Department of Instrumental Analysis, School of Pharmacy with the Division of Laboratory Medicine in Sosnowiec, Medical University of Silesia, Katowice, Poland; ^5^Chair and Department of Physiology, School of Medicine with Dentistry Division in Zabrze, Medical University of Silesia, Katowice, Poland

## Abstract

**Background:**

Equine rhinitis A virus (ERAV) is considered to be an important pathogen in horses, but relatively few studies are available.

**Aims:**

The purpose of this study was to verify ERAV seroprevalence in selected horses in Poland, in addition to correlation between ERAV and age and sex of analysed animals and the antioxidant status.

**Methods:**

The material collected from clinically healthy horses was tested using the VNT (353 serum samples) and virus isolation method (44 nasal swabs). 27 serum samples with antibody titers between 0 and ≥1 : 2048 were chosen for further analysis. The study was conducted in group 1 (ERAV titer ≤ 64) and group 2 (ERAV titer > 64).

**Results:**

Seroneutralisation tests showed positive results in 72% of serum samples. No significant correlation between ERAV seropositive results and selected biochemical indicators was observed. Group 2 had statistically higher concentrations of SOD and CuZnSOD than the analysed group 1.

**Conclusions:**

ERAV was not detected in the nasal swab samples. Antioxidant parameters did not significantly vary between horses of different breed, sex, or age. The ERAV virus had an impact on plasma total SOD and Cu/Zn SOD activity in horses in early stages of convalescence.

## 1. Introduction

Equine rhinitis A virus (ERAV) transmitted through nasal secretions and in urine causes mild to severe upper respiratory tract disease, including fever, anorexia, nasal discharge, bronchitis, pharyngitis, coughing, and swollen lymph nodes [1–4]. Viral respiratory diseases are one of the most costly problems in equine breeding [5]. Owners, practitioners, and virologists, however, usually devote little attention to ERAV infections when compared to herpesviruses' infections and equine influenza. It is believed that ERAV causes a trivial illness, but in many cases this virus weakens the athletic condition of the horse, causing delays in training and also hinders performance at an optimal level for prolonged periods of time [6]. Admittedly, ERAV has been widely isolated and serological investigations in horses have been carried out in many countries, but there is still a lack of detailed information about seroprevalence. The role this virus plays in equine respiratory infections, of a viral nature, has been marginalised, and, as a result, relatively few studies about ERAV infections are available [5, 7]. However, in light of recent research, ERAV is increasingly considered to be an important respiratory pathogen in horses [7]. Reactive oxygen species (ROS) are known to play a role in viral diseases by influencing a host's defensive response. Viral infections influence the production of reactive oxygen species (ROS) in the infected cells and it has been proved that antioxidant systems protect host cells against a variety of viruses by changing their oxidative/antioxidative status [8]. ROS can kill pathogens directly by causing oxidative damage to biocompounds or indirectly by stimulating pathogen elimination through various nonoxidative mechanisms [9]. The changes in biochemical parameters and pathways involved in the generation of ROS during viral infections are not fully understood.

To the best of our knowledge, there have been no serological investigations of equine rhinitis A virus in serum from different equine populations of horses in Poland, to this point. There was also no assessment of the relationship between equine rhinitis A virus infection and antioxidative response in horses. Thus, the overall goal of this study was to determine how many from a group of selected animals in Poland that were commercially used were ERAV seropositive and whether there was any correlation between ERAV and the age or sex of the analysed animals. The second aim of this study was to estimate the antioxidant status in ERAV seropositive horses and whether/how it changes in different stages of convalescence.

## 2. Materials and Methods

Three hundred and fifty-three serum samples were collected from clinically healthy equine populations, originating from all across Poland. The age and gender profile of the selected horses is presented in Table 1. The study included horses from horse riding clubs, breeding farms, purchasing centres, and equine slaughterhouses. The collected material was tested using a virus neutralisation test (VNT) in 96-well microtiter plates. In each well, 25 *μ*l of Minimum Essential Medium (MEM) (Sigma, UK), supplemented with 2% foetal bovine serum (FBS, Sigma, USA), 4 mM glutamine (Sigma, UK), 100 U/ml of penicillin, and 100 *μ*g/ml of streptomycin (Sigma, Germany), was pipetted. Serum samples were inactivated for 30 min at 56°C and then 25 *μ*l was added to a well in the first column to obtain a starting dilution of 1 : 4. From this well, twofold dilutions were made by pipetting 25 *μ*l of each well into the next. Then, 25 *μ*l of hamster complement (Biomed, Lublin, Poland) and finally 25 *μ*l of 100 TCID_50_ of ERAV (V 1722/70, reference strain) were added to each well. Serum and virus were preincubated at 37°C for 1 to 2 hours to allow for neutralisation of the virus. Thereafter, 50 *μ*l of RK-13 cell line suspension per well was added. Plates were then incubated for 5 days at 37°C and under 5% CO_2_. The highest dilution of serum that neutralises the test dose of the virus was determined as the titer of the serum.

### 2.1. Nasal Swabs

**Figure 1:**
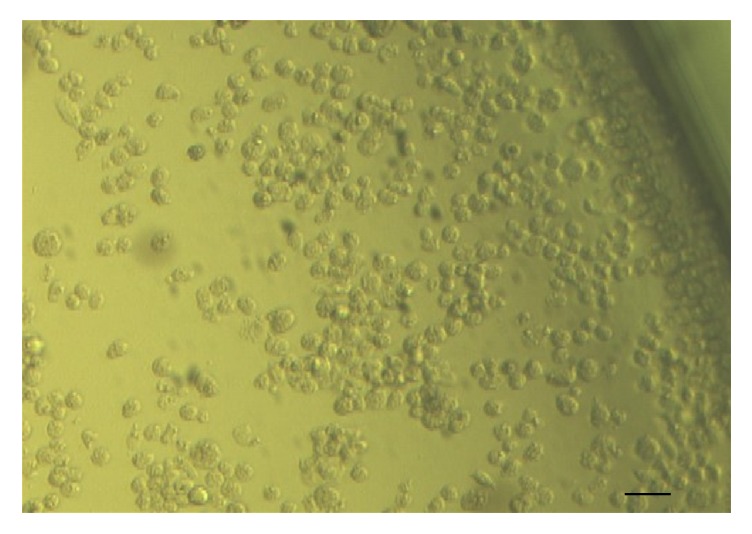
CPE caused by ERAV replication. Visible rounded cells detached from the bottom of the dish and formed aggregates. Scale bar represents 20 *µ*m. Mag. 200x.

**Figure 2:**
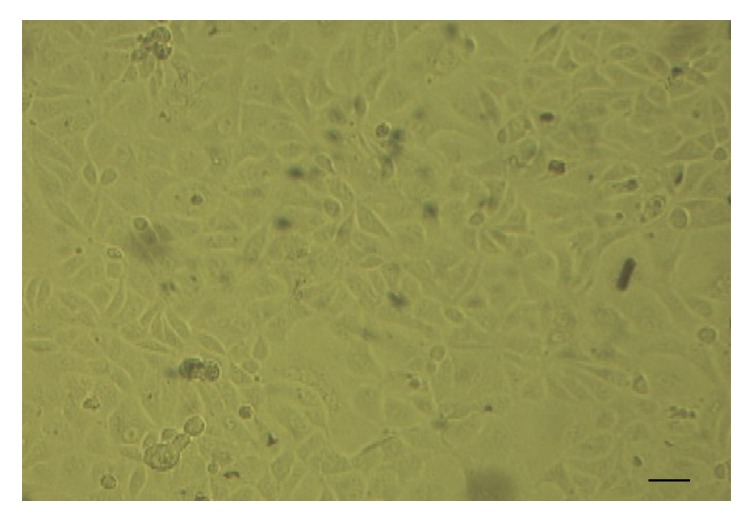
Noninfected RK-13 cell line. Scale bar represents 20 *µ*m. Mag. 200x.

Among the serum samples, 44 were collected together with nasal swabs in order to investigate the presence of ERAV in nasal mucosa. The virus isolation was carried out in an RK-13 (rabbit kidney) cell line. All collected materials were prepared using previously described procedures [10] and then inoculated (50–100 *μ*l per well) into 24-well polystyrene plates containing an RK-13 cell line. The plates were then incubated in 37°C/5% CO_2_ and observed daily for 5–10 days for the development of cytopathic effects (CPE) (Figures 1 and 2), using an inverted microscope (Olympus Corp., Hamburg, Germany; Axio Observer, Carl Zeiss MicroImaging GmbH). In the absence of visible CPE, a second passage was conducted.

### 2.2. Antioxidative Status

Twenty-seven serum samples obtained from the group virological investigated were chosen for biochemical blood research. The antibody titers reached between 0 and ≥1 : 2048. In the case of 16 animals, the titers were between the levels of 1 : 4 and 1 : 64; thus paired sera tests were performed to confirm/exclude the current disease. Titers higher than 1 : 64 or a 4-fold increase in antibody titer obtained in a paired serum samples test were considered to be indicators of recent infection. Based on these assumptions, the investigated horses were divided into two groups: seronegative or infected in the past (titer ≤ 1 : 64) and group of horses which had had recent contact with the virus, had contracted infection shortly before the experiment, or tested positive in a paired serum sample test (titer > 1 : 64).

## 3. Oxidative Stress Markers Analysis

An antioxidant system in the serum was analysed by determining superoxide dismutase (SOD EC 1.15.1.1), ceruloplasmin (CER), the total antioxidant capacity (TAC), and total oxidant status (TOS). Lipid peroxidation was determined by malondialdehyde (MDA) concentration.

### 3.1. Protein Concentration

Protein concentration was determined using the Lowry et al. methods [11].

### 3.2. Superoxide Dismutase Analysis (SOD)

SOD isoenzymes' activity was determined according to the Oyanagui method [12] with KCN as the inhibitor of the CuZnSOD isoenzyme. CuZnSOD activity was calculated as the difference between total SOD activity and MnSOD activity. SOD activity was calculated against a blank probe, containing bidistilled water. Enzyme activity was expressed as nitrite units (NU) per mL serum. One NU exhibits 50% inhibition of the formation of a nitrite ion under the method's condition [12]. The inter- and intra-assay coefficients of variations (CV) were 2.8% and 5.4%, respectively.

### 3.3. Ceruloplasmin

Concentration of CER in serum was determined spectrophotometrically according to Richterich [13] using the reaction with p-phenyl diamine. CER catalyses the oxidation of colourless p-phenylenediamine, replacing it with a blue-violet dye. The absorbance of the samples was read at 560 nm. The measurement was conducted on a PerkinElmer VICTOR-X3 reader. Interassay and intra-assay coefficients of variations were, respectively, 1.3 and 4.0%.

### 3.4. Total Antioxidant Capacity (TAC)

Plasma TAC was measured using a commercial kit (Randox, Co., England). The 2,2′azino-di-(3-ethylbenzothiazoline sulphonate) (ABTS) was incubated with a peroxidase (metmyoglobin) and hydrogen peroxide to produce the radical cation ABTS+, which has a relatively stable blue-green colour and was measured at 600 nm. The suppression of the colour was compared to the standard for TAC measurement assays (Trolox). The assay results are expressed as a Trolox equivalent (mmol/L) [14]. The inter- and intra-assay coefficients of variations (CV) were 1.1% and 3.8%, respectively.

### 3.5. Total Antioxidant Status

The method according to Erel [15] is based on the oxidation of iron (II) ions to iron (III) ions in an acidic medium. Then iron (III) ions with xylene orange form a colourful complex ranging up to a blue-purple colouration. Absorption readings were taken with the 560 nm filter on the VICTOR-X3 from PerkinElmer. The concentration was calculated from the calibration curve using H_2_O_2_ as the standard. Values are expressed in *μ*mol/l.

### 3.6. Lipid Peroxidation

Malondialdehyde (MDA) concentration was measured in samples of serum according to the method described by Ohkawa et al., using the reaction with thiobarbituric acid with spectrophotometric detection, employing 515 nm excitation and 552 nm emission wavelengths. MDA concentration was calculated from the standard curve and prepared from 1,1,3,3-tetraethoxypropane [16]. The inter- and intra-assay coefficients of variations (CV) were 2.1% and 8.3%, respectively.

### 3.7. Lipofuscin (LPS)

Serum levels of LPS were determined according to Tsuchida et al. [17]. Serum was added with 3 : 1 v/v ethanol-ether, shaken, and centrifuged. The intensity of fluorescence was determined using a PerkinElmer spectrophotometer LS45 at a wavelength of 345 nm (absorbance) and 430 nm (emission) in a dissolved solid. The values are expressed in relative lipid extract fluorescence (RF), where 100 RF corresponds to the fluorescence of 0.1 *μ*g/ml quinidine sulphate in 0.1 N sulfuric acid. LPS concentrations are shown in RF. The inter- and intra-assay coefficients of variations (CV) are 2.8% and 9.7%.

## 4. Statistical Analysis

Statistical analysis was performed using STATISTICA 10.0 PL (StatSoft, Cracow, Poland) and StataSE 12.0 (StataCorp LP, TX, USA). Statistical significance was set at a *p* value below 0.05. Data was expressed as means ± SD, and the results were assessed for normality using the Shapiro–Wilk method and the quantile-quantile plot. Correlations among analysed biochemical parameters were assessed using Pearson correlation coefficient. For comparison of selected biochemical parameters, the Student *t*-test was applied.

## 5. Results

### 5.1. Viral and Serological Analyses

Virus isolation tests did not detect ERAV in any of the nasal swab samples. Seroneutralisation tests showed positive results in 254 (72%) serum samples with titer levels from 1 : 4 to 1 : ≥2048. Statistical analysis showed no significant relationship between gender or age and antibodies against ERAV (*p* > 0.05).

### 5.2. Oxidative Status Analysis

**Figure 3:**
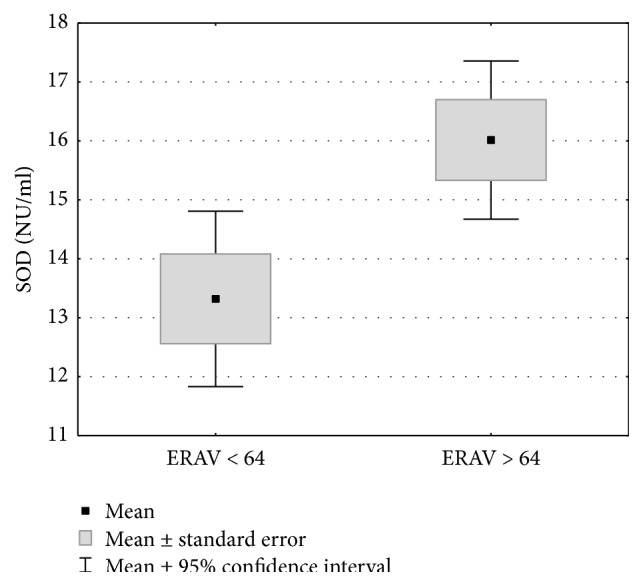
Total superoxide dismutase serum levels of horses from group 1 (ERAV ≤ 64) and group 2 (ERAV > 64). Statistical significance was set at a *p* value below 0.05.

**Figure 4:**
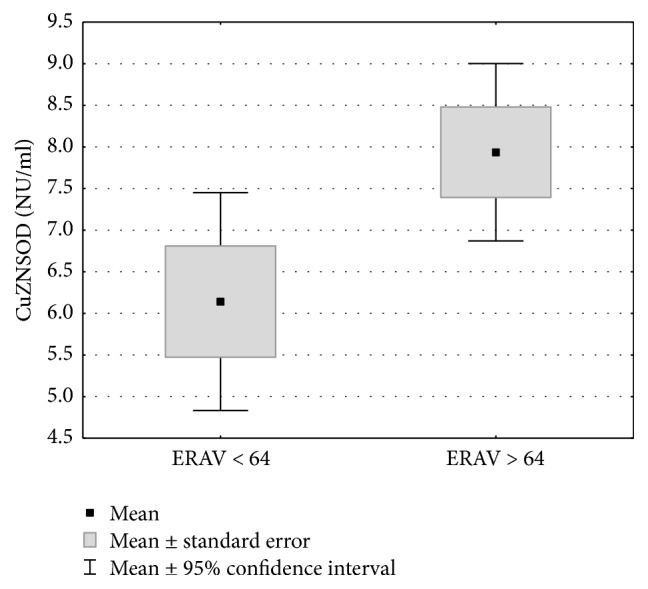
Cu/Zn superoxide dismutase serum levels of horses from group 1 (ERAV ≤ 64) and group 2 (ERAV > 64). Statistical significance was set at a *p* value below 0.05.

The median ERAV titer of 64 was determined and the study group was divided into two subgroups: group 1 (ERAV titer ≤ 64) and group 2 (ERAV titer > 64) (Table 2). There was no statistically significant correlation between ERAV seropositive results and selected biochemical indicators. The high titer group (ERAV > 64) had statistically higher concentrations of SOD and CuZnSOD than the low titer group (*p* < 0.05, Figures 3 and 4 and Table 2). For other biochemical indicators, there were no statistically significant differences between the studied groups (Table 2).

## 6. Discussion

ERAV is commonly found in populations of horses worldwide; nevertheless, to the best of our knowledge, in the populations of horses in the territory of Poland, this virus has not been investigated yet. In this study, 72% of animals were ERAV seropositive, including 46% of horses with serum titer higher than 1 : 64 and 13% of horses with serum titer higher than 1 : 1024, which may suggest that those horses had recently been infected. In general, the studies on ERVs show that the percentages of prevalence may vary from 0% to 100% [18, 19].

In European countries, the prevalence of ERAV was reported as high, at more than 60%. In the United Kingdom, 100% of serologically tested horses had a significant titer to ERAV [19]. Similar percentage of adult seropositive animals (73%) was obtained in Ireland (1998); nevertheless most of the examined horses were febrile [20]. A high prevalence of ERAV was confirmed in Austria in 2005, where 90% of the equine sera and 2.7% of the human sera showed reactivity to ERAV [21], and in France the percentage was 79.4% [22]. In investigations carried out in the United States of America, the prevalence of ERAV neutralising antibodies was reported at the level of 77% [23]. Reports from central-eastern Asia, all provinces of Mongolia, show that this percentage has reached only 34.2% out of a total of 300 horses [24]. According to the authors, this was probably because the blood samples were collected mainly from young horses. Age was also related to the ERAV in the study of Burrows, where 60% of adult horses were seropositive, in comparison with 12.5% of seropositive foals and 8.3% of yearlings [18]. Nevertheless, we did not confirm a significant relationship between age or gender and ERAV antibodies in this study. Unfortunately, we cannot confirm our result concerning the gender, because correlation between sex and ERAV antibodies has not been widely described in the literature on the subject yet.

The prevalence of ERBV, the second equine rhinitis virus belonging to the Picornaviridae family, in equine sera in Poland, is close to 70% [25]. Interestingly, available studies report that ERBV seems to be more commonly isolated than ERAV [7]. In our study, we obtained a similar percentage of ERAV seropositive horses, which means that ERAV and ERBV are equally distributed among the horses in Poland.

The above results have demonstrated that equine rhinoviruses are commonly circulated among horses globally and their clinical importance may have been underestimated [5]. This study shows that ERAV antibodies are highly represented in analysed equine subjects, which suggests that subclinical infections manifested by high seroprevalence in clinically healthy animals are quite possibly a very common occurrence. Nasopharyngeal swabs are best collected from infected horses suspected of being infected within 24–48 hours of infection to detect the virus [7]. In our investigation, the results of virus isolation were negative, probably because the swabs were taken more than two days after infection. Further investigations need to be carried out for a better understanding of pathogenesis and the epidemiology of ERAV virus.

Oxidative stress is considered to play a role in the progression of viral infections [26], contributing to viral replication, inflammation, and decreased immune cell proliferation [27]. It is known that oxidative stress, caused via viral infection, may lead to several aspects of viral pathogenesis, including inflammatory response and viral replication [28]. The oxidant/antioxidant status in different groups of horses is shown to be unbalanced in several physiological situations [29]. The infection by different types of virus results in an imbalanced oxidant/antioxidant relation. The main forces of cell protection against ROS are the enzymatic antioxidant systems (superoxide dismutase, glutathione peroxidase, and catalase) and nonenzymatic antioxidant systems (glutathione, ascorbate, and uric acid), which prevent lipid peroxidation and DNA damage [30]. The strategy of defence against hydroxyl radicals is the dismutation process of superoxide anions (O_2_^−^), conducted by superoxide dismutase, disproportionation of H_2_O_2_ catalysed by CAT, and reduction with GPx [31]. The synthesis of SOD is stimulated by a reduction of molecular oxygen, TNF, interleukins, endotoxins, and chemicals and hypoxia [32, 33]. In our study, horses currently infected by equine rhinitis A virus showed increased serum levels of total SOD and Zn/Cu SOD but not Mn SOD when compared to subjects with past infections or seronegative subjects. Total SOD can be considered as one of the most sensitive markers of oxidative stress [32, 33]. The fact that total SOD increased but not Mn SOD was considered to be a positive trend in the physiological mechanisms of cell protection. Increased total activity of SOD in plasma may be understood as an adaptive response of the cells to elevated oxidative stress markers. Radakovic et al. [34] showed that horses naturally infected by* Theileria equi* present increased erythrocytes levels of Cu/Zn SOD concentrations, which suggests that* T. equi* could have modified isoenzyme activity via free radical production. In our study, the general trend of total oxidant status reduction and lack of a significant increase in MDA serum levels shows the positive effects of antioxidative processes. Other studies show increased levels of blood MDA and decreased antioxidant enzymes, under conditions of stress, in racing horses following jumping activity [35]. On the other hand, Onmaz et al. [36] found that decreased total SOD levels and increased MDA concentrations occur in horses exposed to long-term transport stress, which confirms the presence of independent changes in intra- and extracellular activities of antioxidant systems in response to different stress stimulators and environmental markers.

## 7. Conclusions

ERAV may have an influence on the condition of horses, causing delays in training and hindering performance at an optimal level for prolonged periods of time [6]. ERAV virus antibodies were present in approximately 72% of commercially used horses analysed, which shows that this virus is widely distributed in Poland. The age of horses had no significant effect on antioxidant and virus parameters. The ERAV virus, as a stress marker, had a significant impact on plasma total SOD and Cu/Zn SOD activity in horses in early stages of convalescence when compared with other subjects. A better understanding of the basic physiological processes may enhance the identification of animals at the risk of oxidative stress, which may be accompanied by poor health and has importance for welfare outcomes.

## Figures and Tables

**Table 1:** Age and gender profile of horses included in the study.

	Gender					
Number of animals	Mares		Stallions		Geldings	
in the groups	163		185		5	

	Age (years)					

	1–3 y	4–6 y	7–9 y	10–12	13–15 y	>15 y
Number of animals	83	135	66	39	11	19

Statistical significance was set at a *p* value below 0.05.

**Table 2:** Comparison of analyzed biochemical parameters for group 1 and group 2. Data are expressed as means ± SD. CER, ceruloplasmin; TAC, total antioxidant capacity; TAS, total antioxidant status; SOD, superoxide dismutase; MnSOD, Mn superoxide dismutase; Cu/Zn SOD superoxide dismutase; MDA, malondialdehyde; LPS RF, lipofuscin.

Parameter	Group I ERAV ≤ 64	Group II ERAV > 64	*p*
Protein g/l	68.94 ± 12.37	73.02 ± 7.29	0.311
CER mg/dl	10.86 ± 3.53	10.62 ± 4.55	0.878
TAC mmol/l	0.94 ± 0.11	0.9 ± 0.14	0.441
TOS umol/l	102.4 ± 42.8	83.5 ± 58.7	0.345
*Total SOD NU/ml*	13.32 ± 2.84	16.01 ± 2.47	*<0.05*
Mn SOD NU/ml	7.18 ± 1.78	8.08 ± 2.05	0.234
*Cu/Zn SOD NU/ml*	6.14 ± 2.5	7.94 ± 1.96	*<0.05*
LPS RF	1561 ± 341	1775 ± 448	0.173
MDA umol/l	6.21 ± 3.09	6.59 ± 2.82	0.742

Statistical significance was set at a *p* value below 0.05.
